# Supplementary material for: Meta-Analysis and Gene Set Enrichment Relative to ER Status Reveal Elevated Activity of MYC and E2F in the “Basal” Breast Cancer Subgroup
Source: PLoS One. 2009 Mar 9;4(3):e4710. doi: 10.1371/journal.pone.0004710 (PMC2650420; doi:10.1371/journal.pone.0004710)
Supplement: Dataset S1 — GSEA results from first screen. The file contains a zipped archive of the HTML results of the second GSEA screen. Reports for enriched or depleted datasets may be accessed from the “index.html” link within the my_analysis.GseaPreranked.1219975950625.rpt.zip ZIP archive. Enrichment plots are available for each of the 50 most enriched and depleted gene sets. (13.32 MB ZIP) [file pone.0004710.s001.zip › my_analysis.GseaPreranked.1219975950625.rpt.zip!//CHR12Q13.html]

Details for gene set CHR12Q13[GSEA]

|  || Dataset | zSco.ERneg.to.ERpos\_collapsed |
| Phenotype | NoPhenotypeAvailable |
| Upregulated in class | na\_neg |
| GeneSet | CHR12Q13 |
| Enrichment Score (ES) | -0.45370626 |
| Normalized Enrichment Score (NES) | -2.2653 |
| Nominal p-value | 0.0 |
| FDR q-value | 3.0262212E-4 |
| FWER p-Value | 0.0040 |
Table: GSEA Results Summary

  

Fig 1: Enrichment plot: CHR12Q13      
 Profile of the Running ES Score & Positions of GeneSet Members on the Rank Ordered List

  

| PROBE | GENE SYMBOL | GENE\_TITLE | RANK IN GENE LIST | RANK METRIC SCORE | RUNNING ES | CORE ENRICHMENT || 1 | KRT81 |  |  | 371 | 4.740 | -0.0137 | No |
| 2 | NDUFA4L2 |  |  | 680 | 3.972 | -0.0250 | No |
| 3 | OR7E47P |  |  | 783 | 3.794 | -0.0212 | No |
| 4 | IL23A |  |  | 1176 | 3.264 | -0.0411 | No |
| 5 | SLC16A7 |  |  | 1393 | 3.045 | -0.0482 | No |
| 6 | NXPH4 |  |  | 1407 | 3.037 | -0.0400 | No |
| 7 | INHBC |  |  | 1502 | 2.940 | -0.0381 | No |
| 8 | AQP5 |  |  | 1521 | 2.928 | -0.0306 | No |
| 9 | TROAP |  |  | 1562 | 2.891 | -0.0248 | No |
| 10 | ZBTB39 |  |  | 1588 | 2.863 | -0.0180 | No |
| 11 | B4GALNT1 |  |  | 1874 | 2.627 | -0.0317 | No |
| 12 | DDN |  |  | 1946 | 2.573 | -0.0292 | No |
| 13 | ACVR1B |  |  | 2189 | 2.393 | -0.0403 | No |
| 14 | PPP1R1A |  |  | 2368 | 2.274 | -0.0469 | No |
| 15 | CDK4 |  |  | 2546 | 2.176 | -0.0537 | No |
| 16 | CBX5 |  |  | 2621 | 2.133 | -0.0528 | No |
| 17 | CDK2 |  |  | 2688 | 2.099 | -0.0515 | No |
| 18 | KRT75 |  |  | 2712 | 2.085 | -0.0469 | No |
| 19 | MARS |  |  | 2964 | 1.952 | -0.0600 | No |
| 20 | ADMR |  |  | 3004 | 1.929 | -0.0571 | No |
| 21 | KIAA0286 |  |  | 3022 | 1.923 | -0.0525 | No |
| 22 | ITGB7 |  |  | 3076 | 1.896 | -0.0508 | No |
| 23 | NCKAP1L |  |  | 3194 | 1.845 | -0.0540 | No |
| 24 | AQP6 |  |  | 3204 | 1.837 | -0.0491 | No |
| 25 | RPL41 |  |  | 3471 | 1.722 | -0.0641 | No |
| 26 | WNT1 |  |  | 3495 | 1.712 | -0.0606 | No |
| 27 | MYL6B |  |  | 3506 | 1.705 | -0.0562 | No |
| 28 | LOC57228 |  |  | 3794 | 1.588 | -0.0731 | No |
| 29 | PA2G4 |  |  | 3866 | 1.566 | -0.0738 | No |
| 30 | RBMS2 |  |  | 3955 | 1.531 | -0.0758 | No |
| 31 | CENTG1 |  |  | 4078 | 1.481 | -0.0805 | No |
| 32 | WNT10B |  |  | 4085 | 1.478 | -0.0765 | No |
| 33 | RACGAP1 |  |  | 4097 | 1.475 | -0.0728 | No |
| 34 | DCTN2 |  |  | 4170 | 1.444 | -0.0739 | No |
| 35 | LALBA |  |  | 4236 | 1.417 | -0.0745 | No |
| 36 | METTL7A |  |  | 4266 | 1.408 | -0.0725 | No |
| 37 | K-ALPHA-1 |  |  | 4312 | 1.387 | -0.0717 | No |
| 38 | SLC11A2 |  |  | 4406 | 1.353 | -0.0746 | No |
| 39 | HSU79275 |  |  | 4431 | 1.343 | -0.0723 | No |
| 40 | CYP27B1 |  |  | 4582 | 1.293 | -0.0798 | No |
| 41 | IRAK3 |  |  | 4645 | 1.270 | -0.0806 | No |
| 42 | CNOT2 |  |  | 4674 | 1.260 | -0.0789 | No |
| 43 | MIP |  |  | 4736 | 1.240 | -0.0798 | No |
| 44 | PDE1B |  |  | 4959 | 1.169 | -0.0931 | No |
| 45 | SMARCD1 |  |  | 5045 | 1.138 | -0.0961 | No |
| 46 | KIAA0748 |  |  | 5268 | 1.060 | -0.1097 | No |
| 47 | DTX3 |  |  | 5417 | 1.003 | -0.1179 | No |
| 48 | CCNT1 |  |  | 5647 | 0.931 | -0.1324 | No |
| 49 | RDH5 |  |  | 5747 | 0.897 | -0.1372 | No |
| 50 | IKZF4 |  |  | 5751 | 0.894 | -0.1347 | No |
| 51 | MDM2 |  |  | 5816 | 0.872 | -0.1369 | No |
| 52 | POU6F1 |  |  | 5881 | 0.852 | -0.1392 | No |
| 53 | INHBE |  |  | 5948 | 0.833 | -0.1417 | No |
| 54 | ATP5G2 |  |  | 6071 | 0.791 | -0.1485 | No |
| 55 | SCN8A |  |  | 6076 | 0.789 | -0.1464 | No |
| 56 | FKBP11 |  |  | 6214 | 0.746 | -0.1546 | No |
| 57 | ART4 |  |  | 6362 | 0.697 | -0.1636 | No |
| 58 | PRIM1 |  |  | 6525 | 0.642 | -0.1740 | No |
| 59 | NEUROD4 |  |  | 6550 | 0.634 | -0.1739 | No |
| 60 | SOAT2 |  |  | 6634 | 0.612 | -0.1783 | No |
| 61 | ATF7 |  |  | 6872 | 0.527 | -0.1947 | No |
| 62 | NFE2 |  |  | 6887 | 0.523 | -0.1942 | No |
| 63 | TAC3 |  |  | 6950 | 0.503 | -0.1973 | No |
| 64 | ITGA7 |  |  | 7035 | 0.475 | -0.2023 | No |
| 65 | RARG |  |  | 7070 | 0.461 | -0.2035 | No |
| 66 | SLC38A4 |  |  | 7399 | 0.346 | -0.2273 | No |
| 67 | KIF5A |  |  | 7409 | 0.343 | -0.2270 | No |
| 68 | P11 |  |  | 7452 | 0.328 | -0.2292 | No |
| 69 | GRIP1 |  |  | 7790 | 0.192 | -0.2542 | No |
| 70 | BAZ2A |  |  | 7968 | 0.117 | -0.2672 | No |
| 71 | NR4A1 |  |  | 8011 | 0.101 | -0.2701 | No |
| 72 | MYO1A |  |  | 8072 | 0.080 | -0.2744 | No |
| 73 | FAIM2 |  |  | 8087 | 0.076 | -0.2753 | No |
| 74 | GDF11 |  |  | 8517 | -0.087 | -0.3076 | No |
| 75 | NPFF |  |  | 8518 | -0.087 | -0.3073 | No |
| 76 | CIP29 |  |  | 8554 | -0.103 | -0.3097 | No |
| 77 | AVIL |  |  | 8685 | -0.163 | -0.3191 | No |
| 78 | SILV |  |  | 8760 | -0.191 | -0.3241 | No |
| 79 | APOF |  |  | 8821 | -0.219 | -0.3280 | No |
| 80 | KRT8 |  |  | 8956 | -0.291 | -0.3373 | No |
| 81 | AMHR2 |  |  | 8961 | -0.292 | -0.3367 | No |
| 82 | LARP4 |  |  | 8967 | -0.297 | -0.3362 | No |
| 83 | ATP5B |  |  | 9027 | -0.325 | -0.3397 | No |
| 84 | KRT84 |  |  | 9082 | -0.352 | -0.3427 | No |
| 85 | CS |  |  | 9107 | -0.365 | -0.3434 | No |
| 86 | KRT76 |  |  | 9317 | -0.464 | -0.3579 | No |
| 87 | LEPREL2 |  |  | 9402 | -0.510 | -0.3627 | No |
| 88 | FLJ13236 |  |  | 9465 | -0.545 | -0.3657 | No |
| 89 | SLC26A10 |  |  | 9669 | -0.655 | -0.3792 | No |
| 90 | GLI1 |  |  | 9714 | -0.676 | -0.3804 | No |
| 91 | ERBB3 |  |  | 9745 | -0.694 | -0.3806 | No |
| 92 | DDIT3 |  |  | 9789 | -0.718 | -0.3817 | No |
| 93 | TFCP2 |  |  | 9836 | -0.747 | -0.3829 | No |
| 94 | DGKA |  |  | 9842 | -0.751 | -0.3810 | No |
| 95 | LRP1 |  |  | 10000 | -0.845 | -0.3903 | No |
| 96 | RDH16 |  |  | 10181 | -0.944 | -0.4011 | No |
| 97 | GLS2 |  |  | 10352 | -1.064 | -0.4108 | No |
| 98 | SP1 |  |  | 10401 | -1.092 | -0.4111 | No |
| 99 | HNRPA1 |  |  | 10609 | -1.250 | -0.4231 | No |
| 100 | METTL1 |  |  | 10644 | -1.279 | -0.4217 | No |
| 101 | ARF3 |  |  | 11055 | -1.622 | -0.4480 | No |
| 102 | FLJ20489 |  |  | 11097 | -1.660 | -0.4460 | No |
| 103 | NAB2 |  |  | 11191 | -1.737 | -0.4478 | No |
| 104 | FLJ21908 |  |  | 11270 | -1.805 | -0.4482 | Yes |
| 105 | SPATS2 |  |  | 11318 | -1.841 | -0.4462 | Yes |
| 106 | YEATS4 |  |  | 11327 | -1.848 | -0.4412 | Yes |
| 107 | HDAC7A |  |  | 11335 | -1.861 | -0.4360 | Yes |
| 108 | OBFC2B |  |  | 11372 | -1.899 | -0.4330 | Yes |
| 109 | PTGES3 |  |  | 11463 | -2.000 | -0.4337 | Yes |
| 110 | RNF41 |  |  | 11501 | -2.037 | -0.4303 | Yes |
| 111 | PCBP2 |  |  | 11508 | -2.045 | -0.4246 | Yes |
| 112 | HSD17B6 |  |  | 11578 | -2.108 | -0.4234 | Yes |
| 113 | CACNB3 |  |  | 11624 | -2.146 | -0.4203 | Yes |
| 114 | CTDSP2 |  |  | 11625 | -2.146 | -0.4137 | Yes |
| 115 | C12ORF44 |  |  | 11660 | -2.179 | -0.4097 | Yes |
| 116 | LMBR1L |  |  | 11758 | -2.274 | -0.4101 | Yes |
| 117 | MYL6 |  |  | 11843 | -2.362 | -0.4093 | Yes |
| 118 | USP52 |  |  | 11931 | -2.478 | -0.4084 | Yes |
| 119 | EIF4B |  |  | 11934 | -2.480 | -0.4010 | Yes |
| 120 | TMEM106C |  |  | 12224 | -2.877 | -0.4142 | Yes |
| 121 | IGFBP6 |  |  | 12398 | -3.098 | -0.4179 | Yes |
| 122 | STAT6 |  |  | 12446 | -3.173 | -0.4118 | Yes |
| 123 | LETMD1 |  |  | 12467 | -3.211 | -0.4035 | Yes |
| 124 | SMUG1 |  |  | 12520 | -3.294 | -0.3974 | Yes |
| 125 | RAPGEF3 |  |  | 12567 | -3.365 | -0.3907 | Yes |
| 126 | NELL2 |  |  | 12593 | -3.420 | -0.3822 | Yes |
| 127 | AAAS |  |  | 12729 | -3.686 | -0.3812 | Yes |
| 128 | CALCOCO1 |  |  | 12786 | -3.834 | -0.3738 | Yes |
| 129 | PIP5K2C |  |  | 12796 | -3.883 | -0.3626 | Yes |
| 130 | MCRS1 |  |  | 12800 | -3.891 | -0.3510 | Yes |
| 131 | FAM62A |  |  | 12824 | -3.940 | -0.3408 | Yes |
| 132 | ATF1 |  |  | 12856 | -4.022 | -0.3309 | Yes |
| 133 | C12ORF41 |  |  | 12903 | -4.160 | -0.3217 | Yes |
| 134 | R3HDM2 |  |  | 12913 | -4.187 | -0.3096 | Yes |
| 135 | MAP3K12 |  |  | 12914 | -4.187 | -0.2969 | Yes |
| 136 | AMIGO2 |  |  | 12988 | -4.428 | -0.2889 | Yes |
| 137 | COPZ1 |  |  | 13011 | -4.495 | -0.2769 | Yes |
| 138 | BLOC1S1 |  |  | 13032 | -4.559 | -0.2646 | Yes |
| 139 | TSPAN31 |  |  | 13033 | -4.568 | -0.2507 | Yes |
| 140 | OS9 |  |  | 13038 | -4.597 | -0.2370 | Yes |
| 141 | PRO1843 |  |  | 13068 | -4.718 | -0.2248 | Yes |
| 142 | DDX23 |  |  | 13079 | -4.749 | -0.2111 | Yes |
| 143 | SLC4A8 |  |  | 13080 | -4.751 | -0.1966 | Yes |
| 144 | TENC1 |  |  | 13098 | -4.809 | -0.1833 | Yes |
| 145 | CSAD |  |  | 13100 | -4.817 | -0.1687 | Yes |
| 146 | C12ORF22 |  |  | 13181 | -5.249 | -0.1588 | Yes |
| 147 | ASB8 |  |  | 13208 | -5.446 | -0.1442 | Yes |
| 148 | SUOX |  |  | 13215 | -5.537 | -0.1278 | Yes |
| 149 | C12ORF10 |  |  | 13236 | -5.737 | -0.1118 | Yes |
| 150 | RAB5B |  |  | 13237 | -5.738 | -0.0944 | Yes |
| 151 | PRR13 |  |  | 13269 | -6.207 | -0.0778 | Yes |
| 152 | LIMA1 |  |  | 13275 | -6.263 | -0.0591 | Yes |
| 153 | GALNT6 |  |  | 13281 | -6.334 | -0.0402 | Yes |
| 154 | MFSD5 |  |  | 13293 | -6.481 | -0.0213 | Yes |
| 155 | KRT18 |  |  | 13317 | -7.649 | 0.0002 | Yes |
Table: GSEA details [plain text format]

  

Fig 2: CHR12Q13: Random ES distribution      
 Gene set null distribution of ES for **CHR12Q13**
